# Effects of Prolonged Type 2 Diabetes on the Inner Retinal Layer and Macular Microvasculature: An Optical Coherence Tomography Angiography Study

**DOI:** 10.3390/jcm9061849

**Published:** 2020-06-13

**Authors:** Min-Woo Lee, Woo-Hyuk Lee, Cheon-Kuk Ryu, Tae-Yeon Kim, Hyung-Bin Lim, Young-Hoon Lee, Jung-Yeul Kim

**Affiliations:** 1 Department of Ophthalmology, Konyang University College of Medicine, Daejeon 35365, Korea; bogus1105@gmail.com (M.-W.L.); skykty96@kyuh.ac.kr (T.-Y.K.); astrix001@gmail.com (Y.-H.L.); 2 Department of Ophthalmology, Chungnam National University College of Medicine, Daejeon 35015, Korea; lwhyuk@naver.com (W.-H.L.); chkryu6@gmail.com (C.-K.R.); cromfans@hanmail.net (H.-B.L.); 3 Department of Ophthalmology, Chungnam National University Hospital, #640 Daesa-dong, Jung-gu, Daejeon 301-721, Korea

**Keywords:** diabetes, optical coherence tomography angiography, ganglion cell-inner plexiform layer, vessel density

## Abstract

Purpose: To identify the effects of prolonged type 2 diabetes (T2DM) on macular microcirculation and the inner retinal layer in diabetic eyes without clinical diabetic retinopathy (DR). Methods: 97, 92, and 57 eyes in the control, patients with T2DM < 10 years (DM group one), and patients with T2DM ≥ 10 years (DM group two) were enrolled. The ganglion cell-inner plexiform layer (GC-IPL) thickness and superficial vessel density (VD) were compared. Linear regression analyses were performed to identify factors associated with VD in T2DM patients. Results: GC-IPL thicknesses in the control, DM group one, and DM group two were 84.58 ± 0.89, 83.49 ± 0.70, and 79.04 ± 0.96 μm, respectively (*p* < 0.001). The VDs of the full area were 20.32 ± 0.15, 19.46 ± 0.17, and 18.46 ± 0.23 mm^−1^ (*p* < 0.001). Post-hoc analyses revealed that the VDs of the full area was significantly different in the control vs. DM group one (*p* = 0.001), control vs. DM group two (*p* < 0.001), and DM group one vs. DM group two (*p* = 0.001). Multivariate linear regression analyses revealed that DM duration (*p* = 0.037), visual acuity (*p* = 0.013), and GC-IPL thickness (*p* < 0.001) were significantly associated with the VD of T2DM patients. Conclusions: We confirmed GC-IPL thinning and decreased superficial VD in the macular areas using OCTA in T2DM patients. Patients with T2DM ≥ 10 years exhibited significantly more severe macular microcirculation impairment compared to patients with T2DM < 10 years and normal controls.

## 1. Introduction

Type 2 diabetes (T2DM) is a major global health problem that is increasing in prevalence due to lifestyle changes [1,2,3,4]. The International Diabetes Federation estimated that there were 451 million people with diabetes worldwide in 2017, and expected to increase to 693 million by 2045 [4]. This increase in T2DM could cause an increase in diabetic retinopathy (DR) [5]. DR, which is the leading cause of blindness in the working-age population, is known to be associated with ischemic damage on retina following to change in the microvasculature [6,7,8]. Such retinal damage is also found in patients without clinical DR. Previous studies have found that retinal damage caused by T2DM could result in thinning of the ganglion cell-inner plexiform layer (GC-IPL) and peripapillary retinal nerve fiber layer (pRNFL) in T2DM patients without clinical DR [9,10,11]. Such inner retinal thinning reflects diabetic retinal neurodegeneration, an early event in DR pathogenesis activated by pathologic pathways such as polyols, hexosamine, or oxidative stress [12,13]. Recently, with the development of devices that are able to observe retinal microvasculature and retinal perfusion in detail, various studies on retinal microvasculature damage in patients with T2DM have been reported. 

Optical coherence tomography angiography (OCTA) is a noninvasive imaging technique used to examine the microvasculature of the retina and choroid, which enables visualization of the fine vasculatures of multiple layers. Several studies using OCTA have reported reduced macular microcirculation in patients with T2DM [14,15,16,17]. Cao et al. [14] found that the parafoveal vessel density (VD) of both the superficial and deep capillary plexuses was decreased in diabetic eyes without clinical DR. Li et al. [15] reported foveal avascular zone (FAZ) enlargement and an increased FAZ perimeter in T2DM patients without clinical DR compared to normal controls. As such, retina can be damaged by T2DM in various forms and cannot be concluded to be a normal state even if clinical DR is not observed. Therefore, the state of the retina without clinical DR might be different depending on various factors, but few studies have explored how T2DM duration affects retinal microvasculature.

The purpose of this study was to identify the effects of prolonged T2DM on macular microvasculature and the inner retinal layer by comparing the superficial VD and GC-IPL thickness of patients with T2DM < 10 years and patients with T2DM ≥ 10 years.

## 2. Methods

### 2.1. Patients

This observational, cross-sectional study adhered to the tenets of the Declaration of Helsinki and was approved by the Institutional Review Board of Chungnam National University Hospital, Daejeon, Republic of Korea. We reviewed the charts of patients with T2DM who visited the retina clinic of Chungnam National University Hospital for DR checkups from March 2017 to December 2019. We recorded detailed histories and best-corrected visual acuity (BCVA), intraocular pressure (IOP), spherical equivalent (SE), and axial length (using an IOLMaster; Carl Zeiss, Jena, Germany, version 5.02). Subjects were divided into three groups: Control, patients with T2DM < 10 years (DM group one), and patients with T2DM ≥ 10 years (DM group two). Exclusion criteria were a history of systemic disease other than T2DM; any ophthalmic disease such as glaucoma, retinal diseases, or neuro-ophthalmic diseases; axial length ≥ 26.0 mm; any prior intraocular surgery except cataract extraction; a BCVA < 0.7; and an IOP > 21 mmHg. We also excluded patients with clinical evidence of DR such as retinal hemorrhage or microaneurysms. One eye was randomly selected in patients satisfied with inclusion criteria.

### 2.2. OCT Measurements

We performed SD-OCT (Cirrus HD OCT 5000, version 10.0; Carl Zeiss Meditec, Dublin, CA, USA) using 512 by 128 macular cube and 200 by 200 optic disc cube scanning protocols to measure GC-IPL and pRNFL thicknesses. The ganglion cell analysis algorithm automatically measured GC-IPL thickness by identifying the outer boundaries of the RNFL and the IPL of the macula using the three-dimensional information from the macular cube scan. The average, minimum, and six sectoral (superior, superonasal, inferonasal, inferior, inferotemporal, and superotemporal) GC-IPL thicknesses were analyzed. Images with a signal strength < 7, obvious decentration, or segmentation errors were excluded.

### 2.3. VD Measurement Using OCTA

**Figure 1:**
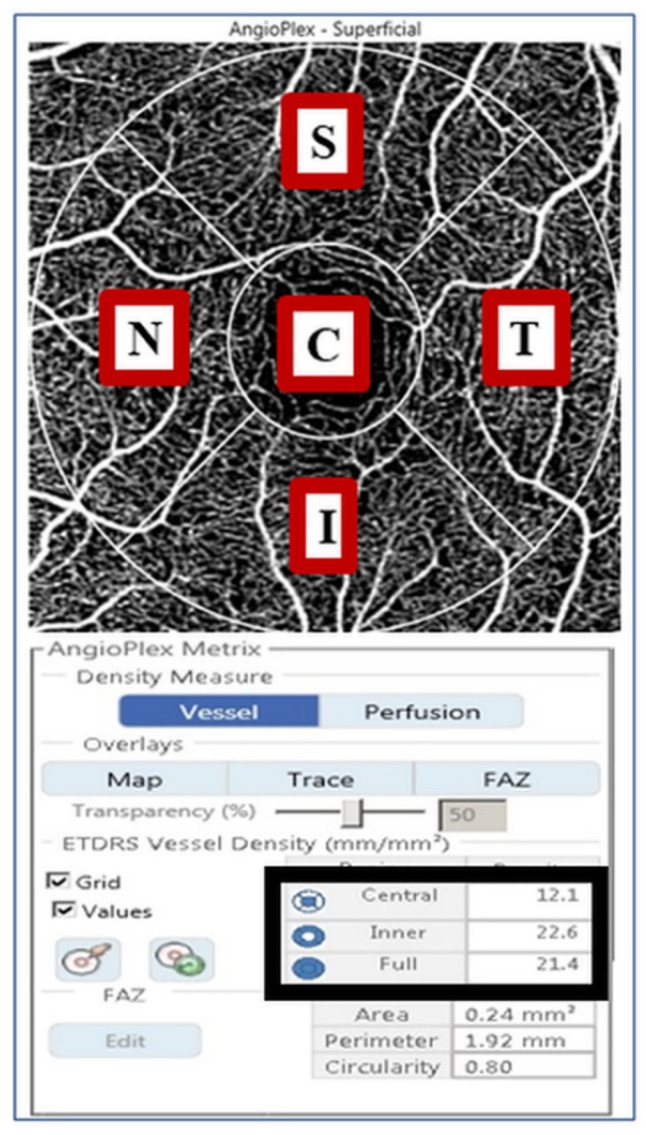
A 3 by 3 mm optical coherence tomography angiography image centered on the fovea. The en face image of the superficial layer is overlaid with the Early Treatment of Diabetic Retinopathy Study grid. The measurement tool (AngioPlex software, V. 10.0; Carl Zeiss Meditec) yields vessel density measurements in individual subfields. The black box contains quantitative vessel density measurements of the central, inner, and full areas. C, center; S, superior; T, temporal; I, inferior; N, nasal.

OCTA images were obtained by an experienced examiner using a Cirrus HD-OCT 5000 along with AngioPlex software (Carl Zeiss Meditec). AngioPlex yields high-resolution retinal microvascular images using a center wavelength of 840 nm, taking 68,000 A-scans per second. The instrument provides sensitivity and accuracy by incorporating the optical microangiography (OMAG) algorithm and retinal tracking technology. We obtained foveal centered scan area of 3 by 3 mm pattern, and all scans were analyzed using en face OCTA images generated automatically by the OMAG algorithm used in AngioPlex software. The VD of the superficial capillary plexus, which spanned from the internal limiting membrane to the IPL, was measured automatically by the software. The software quantified VD via the Early Treatment of Diabetic Retinopathy Study subfields (Figure 1). 

All images were checked and verified by two blinded observers (M.W.L. and W.H.L.), and any images exhibiting fixation loss, segmentation errors, motion artifacts, or signal strengths <8 were excluded.

### 2.4. Statistical Analysis

Demographic characteristics and ocular parameters were compared via one-way analysis of variance with the post-hoc Bonferroni correction and the chi-squared test. Univariate and multivariate linear regression analyses were performed to identify factors associated with superficial macular VD in patients with T2DM. All statistical analyses were performed with the aid of SPSS software (version 18.0; IBM Corp., Armonk, NY, USA).

## 3. Results

### 3.1. Demographics

A total of 246 eyes were enrolled: 97 in the control group, 92 in DM group one, and 57 in DM group two. The mean ages of each group were 63.82 ± 0.44, 62.43 ± 1.09, and 64.12 ± 1.11 years, respectively, which did not show a significant difference (*p* = 0.356) (Table 1). 

Sex, spherical equivalent, IOP, and axial length were not also significantly different among the groups. Visual acuity showed a significant difference among the groups (*p* = 0.027), but this difference disappeared on post-hoc analyses (control vs. DM group one, *p* = 0.056; control vs. DM group two, *p* = 0.088; DM group one vs. DM group two, *p* = 1.000). The T2DM durations were 3.51 ± 0.29 and 14.61 ± 0.57 years (*p* < 0.001), and the HbA1c were 6.90 ± 0.10 and 7.04 ± 0.12% (*p* = 0.397) in DM group one and DM group two, respectively.

### 3.2. GC-IPL Thickness in Each Group

Average GC-IPL thicknesses in the control group, DM group one, and DM group two were 84.58 ± 0.89, 83.49 ± 0.70, and 79.04 ± 0.96 μm, respectively, which showed a significant difference (*p* < 0.001) (Table 2). 

On post-hoc analyses, significant differences were observed in the control vs. DM group two, and DM group one vs. DM group two (*p* < 0.001 and *p* = 0.002, respectively). Additionally, the GC-IPL thicknesses of all six sectors differed significantly among the groups. On post-hoc analyses, control vs. DM group two and DM group one vs. DM group two were significantly different as same as the result of comparison in the average GC-IPL thickness.

### 3.3. Superficial Macular VD in Each Group

**Figure 2:**
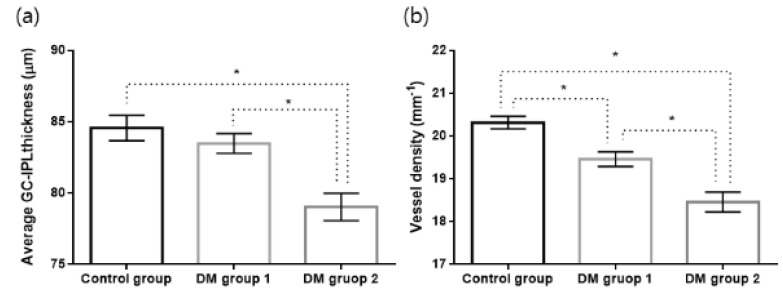
Bar graph with standard errors of average ganglion cell-inner plexiform layer (GC-IPL) thickness (**a**) and vessel density of 3 mm full area (**b**) in each group. * Statistically significant difference. DM group 1, patients with type 2 diabetes < 10 years; DM group 2, patients with type 2 diabetes ≥ 10 years.

The average signal strengths of OCTA images were 9.71 ± 0.05, 9.67 ± 0.05, and 9.61 ± 0.08 in the control, DM group one, and DM group two, respectively (*p* = 0.165). The VDs of the full areas were 20.32 ± 0.15, 19.46 ± 0.17, and 18.46 ± 0.23 mm^−1^ in the control, DM group one, and DM group two, respectively, which showed a significant difference (*p* < 0.001) (Table 3) (Figure 2). 

The VDs of the inner, and central areas and four sectors also differed significantly. On post-hoc analyses, the VDs of the full, and inner areas, and four sectors were significantly different in the control vs. DM group one, control vs. DM group two, and DM group one vs. DM group two.

### 3.4. Factors Associated with Superficial Macular VD in T2DM Patients

**Figure 3:**
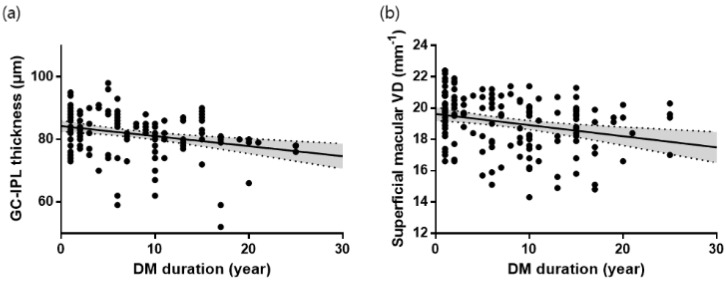
Scatterplots and linear regression analyses between the duration of type 2 diabetes (DM) and GC-IPL thickness (*r* = 0.284, *p* < 0.001) (**a**) and superficial macular vessel density (VD) (*r* = 0.259, *p* = 0.001) (**b**) in patients with DM.

On univariate linear regression analyses, the duration of T2DM (B = −0.07, *p* = 0.002), visual acuity (B = −5.13, *p* = 0.028), and the average GC-IPL thickness (B = 0.08, *p* < 0.001) were significant factors associated with the full VD of macular area (Table 4, Figure 3).

**Figure 4:**
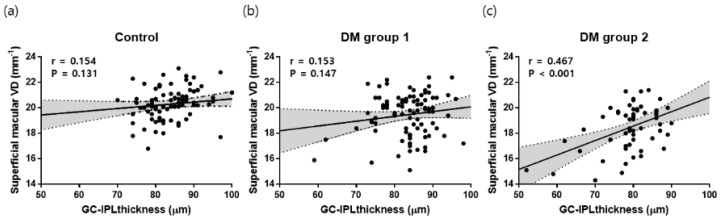
Scatterplots and linear regression analyses between superficial macular VD and GC-IPL thickness for each group. (**a**) control group, (**b**) DM group 1, (**c**) DM group 2. DM group 1 = patients with type 2 diabetes < 10 years, DM group 2 = patients with type 2 diabetes ≥ 10 years.

Multivariate analyses revealed that T2DM duration (B = −0.05, *p* = 0.037), visual acuity (B = −5.39, *p* = 0.013), and average GC-IPL thickness (B = 0.07, *p* < 0.001) were significant factors, in agreement with the univariate analyses (Figure 4).

## 4. Discussion

Previous studies have reported reduced retinal blood circulation times and oxygen maldistributions in the major peripapillary arteries and veins of T2DM patients without DR. [18,19] Others have found that some diabetic eyes without any visible microvascular findings on ophthalmoscopy exhibited microaneurysms on OCTA images [14,20]. Additionally, diabetic retinal neurodegeneration can cause inner retinal thinning and delayed implicit time of multifocal electroretinography prior to DR development [12,13,21]. As such, retinal and circulatory damage caused by T2DM may present in various forms and to various extents even in eyes without clinical DR. We focused on the GC-IPL and microvasculature of the macular area, the most important area on vision, and evaluated the effects of prolonged T2DM on macular microvasculature in eyes without clinical DR. We observed thinning of GC-IPL and reduced superficial VD of the macular area in patients with T2DM, and the patients with prolonged DM had more prominent damage. Additionally, DM duration, visual acuity, and GC-IPL thickness were significantly associated with superficial macular VD in T2DM patients. 

Previous studies have reported GC-IPL damage in patients with T2DM [22,23,24]. Ng et al. [22] reported that subjects with T2DM but without DR had an average GC-IPL thinning of 4.37 μm, compared to controls. Lim et al. [23] found that the estimated reduction rate of the average GC-IPL thickness in T2DM patients without DR (−0.627 μm/year) was 2.26-fold faster than that in normal individuals (−0.277 μm/year). We obtained similar results: The GC-IPL was significantly thinner in T2DM patients than controls. In patients with T2DM, diabetic retinal neurodegeneration, which may develop before any definite microvascular change as mentioned above, manifests as neuronal apoptosis and reactive gliosis. Retinal ganglion and amacrine cells are known to be the first neurons to exhibit diabetes-induced apoptosis, which could result in thinning of the inner retinal layers including the GC-IPL before the prominent retinal vascular changes of DR are apparent [11,13,25]. Such thinning could indirectly provide information on central nervous system (CNS) damage caused by T2DM, which is an easily accessible and non-invasive way. The retina is ontogenically brain-derived tissue, and diabetic retinal neurodegeneration and neurodegenerative diseases such as Alzheimer’s disease are known to share several pathogenic pathways, such as insulin signaling impairment, the accumulation of advanced glycation end-products, and an increase in oxidative stress [26]. Therefore, even without DR, GC-IPL monitoring, which could provide information on CNS damage as well as retina, is crucial especially to patients with prolonged T2DM patients. 

We observed a decreased superficial macular VD in T2DM patients. Zeng et al. [16] identified significantly decreased parafoveal and perifoveal VDs of both the superficial and deep capillary plexus in T2DM patients without DR, compared to controls. Alibhai et al. [17] reported a significantly larger FAZ area and lower binarized flow index of both the superficial and deep retinal layers in diabetic eyes without DR, compared to normal controls. In the retina, neuronal, glial, and vascular cells are closely connected in the neurovascular unit, and an autoregulatory response of the neurovascular unit to complex circulatory and neural cues is essential to regulate blood flow through the inner retina because of lacking autonomic innervation of the intra-retinal vasculature [27,28]. Therefore, the decreased VD may be affected by the disruption of neurovascular autoregulation which could get damaged by diabetic retinal neurodegeneration, but further studies to prove the direct relationship between autoregulation and macular VD are needed to confirm this [13,29]. Additionally, endothelial cell injuries by chronic hyperglycemia can cause acellular retinal capillaries, which may result in reduced macular VD [13,16]. Thus, T2DM patients, even those lacking clinical DR, would have impaired macular microvasculature. 

The superficial macular VDs differed significantly between DM group one and DM group two, and DM duration was significantly associated with the superficial macular VD of T2DM patients. Whereas, HbA1C was not significantly related to superficial macular VD. Sohn et al. [30] reported that the progressive neuroretinal degeneration was primarily related to DM duration and not to HbA1C. Diabetic retinal neurodegeneration is closely related to macular microvasculature by the neurovascular unit, which may result in the appearance of a similar trend. Although clinical information on the relationship between glycemic control and retinal neurodegeneration and macular microvasculature is not available, the major components of the renin-angiotensin system have been identified to be overexpressed in the retina of DM patients, and the blockade of the system in experimental models of DM attenuated retinal neurodegeneration [31,32]. Thus, even under the relatively good glycemic control, prolonged DM may exacerbate macular microvasculature impairment. So, physicians should not conclude that the retina of DM patients is under a stable and safe state even they are not showing DR and under good glycemic control.

Samara et al. [33] found a positive correlation of VD with visual acuity at the level of both the superficial and deep vascular networks in patients with DR. Li et al. [15] reported that logMAR BCVA and FAZ showed a significant negative correlation with superficial VD in DM patients without DR, suggesting that the FAZ and superficial VD are potentially sensitive factors that affect the visual acuity in DM subjects. Our study also found that visual acuity was significantly associated with superficial macular VD in T2DM patients. In the situation of impairment of choroidal circulation due to failure of the autoregulatory mechanism in DM, the metabolic demands of the outer retina including photoreceptor would become more dependent on the vascular supply from the inner retina, which might directly link superficial macular VD to visual acuity [34]. Impairment of vision-related quality of life in T2DM patients such as decreased hue discrimination, decreased contrast sensitivity, delayed dark adaptation, and reduced visual field sensitivity, which was identified to the consequences of diabetic retinal neurodegeneration, would be also related to macular microvasculature impairment, although further studies are needed to confirm this [21,35,36,37]. Besides a promising tool for monitoring and follow-up of macular ischemia, superficial macular VD could be one of the significant factors reflecting the visual function in patients with DM. 

Vujosevic et al. [38] found a significant correlation between OCTA parameters such as perfusion density or VD and RNFL thickness in the peripapillary area. Mase et al. [39] explained this correlation that radial peripapillary plexus is the most important structure in maintaining RNFL integrity. We also found that the average GC-IPL thickness correlated significantly with the superficial macular VD, but only in DM group two. Notably, Lim et al. [40] reported that the average GC-IPL thickness was significantly correlated with the superficial macular VD only in subjects with hypertension ≥ 10 years. The ganglion cell bodies in the macular area are multilayered, and are 10- to 20-fold thicker than their axons [41]. Additionally, whereas the peripapillary area consists of capillaries with long straight paths and rare anastomotic connections, the superficial capillary plexus of the macula has a dense capillary network with numerous anastomoses, which may compensate to some extent for hypoxic damage caused by systemic disease [42,43,44]. Therefore, enough time for damages by diabetic retinal neurodegeneration of ganglion cell bodies in the macular area might be needed before a direct relationship between GC-IPL and superficial macular VD is evident [45]. 

Our study had several limitations. First, the retrospective nature of the work inevitably introduces some selection bias. Second, we did not analyze the VD of the deep capillary plexus because the automatic AngioPlex microcirculation parameters pertain only to the superficial capillary plexus. However, analysis of the superficial capillary plexus is more accurate than that of the deep capillary plexus because of the projection artifacts almost occurring in current technology [46]. Third, as subjects with T2DM ≥ 10 years had a higher probability of having DR or other systemic diseases than other groups, there were fewer cases in DM group two meeting the inclusion criteria than other groups. The strength of our study was that we enrolled OCTA images with SS ≥ 8, allowing accurate analyses. Likewise, this is the first study to identify the impact of prolonged DM on the macular microvasculature besides the inner retinal layer by dividing groups according to the DM duration. 

## 5. Conclusions

In conclusion, we confirmed GC-IPL thinning and decreased superficial VD on the macular areas using OCTA in T2DM patients without clinical DR. Patients with T2DM ≥ 10 years exhibited significantly more severe macular microvasculature impairment than T2DM patients with relatively short disease durations or normal controls, even they were under relatively good glycemic control. Even in the absence of clinical DR, physicians should consider constant diabetic retinal neurodegeneration and macular microvasculature impairment in T2DM patients, and monitor GC-IPL and macular VD using OCTA.

## Figures and Tables

**Table 1:** Demographics and clinical characteristics.

	Normal Controls (*n* = 97)	DM Group 1 (*n* = 92)	DM Group 2 (*n* = 57)	*p*-Value
Age (mean ± SE, years)	63.82 ± 0.44	62.43 ± 1.09	64.12 ± 1.11	0.356
Sex (male, %)	44 (45.4%)	35 (38.0%)	28 (49.1%)	0.371
Laterality (right, %)	55 (56.7%)	46 (50.0%)	28 (49.1%)	0.555
BCVA (mean ± SE, logMAR)	−0.019 ± 0.006	0.002 ± 0.007	0.003 ± 0.007	0.027
SE (mean ± SE, diopters)	0.06 ± 0.11	−0.36 ± 0.14	0.07 ± 0.18	0.060
IOP (mean ± SE, mmHg)	15.64 ± 0.27	15.96 ± 0.32	15.47 ± 0.36	0.600
Axial length (mean ± SE, mm)	23.57 ± 0.07	23.72 ± 0.08	23.51 ± 0.11	0.196
DM duration (mean ± SE, years)	0	3.51 ± 0.29	14.61 ± 0.57	<0.001
HbA1C (mean ± SE, %)	N/A	6.90 ± 0.10	7.04 ± 0.12	0.397
CMT (mean ± SE, μm)	250.22 ± 1.99	246.04 ± 1.93	248.49 ± 2.44	0.317
pRNFL thickness (mean ± SE, μm)	96.71 ± 0.88	94.82 ± 1.07	92.49 ± 1.34	0.032

SE = standard errors; BCVA = best-corrected visual acuity; SE = spherical equivalent; IOP = intraocular pressure; DM = diabetes; CMT = central macular thickness; and pRNFL = peripapillary retinal nerve fiber layer. DM group 1 = patients with type 2 diabetes < 10 years, DM group 2 = patients with type 2 diabetes ≥ 10 years. Values in boldface (*p* < 0.05) are statistically significant.

**Table 2:** Ganglion cell-inner plexiform layer thickness in each group.

	Control	DM Group 1	DM Group 2	*p*-Value
Average	84.58 ± 0.89	83.49 ± 0.70	79.04 ± 0.96	<0.001
Minimum	79.61 ± 0.91	80.03 ± 0.94	73.39 ± 1.70	<0.001
Sector				
Superior	83.89 ± 0.82	84.39 ± 0.71	78.98 ± 1.11	<0.001
Superotemporal	82.13 ± 1.03	81.91 ± 0.78	78.81 ± 1.03	0.014
Inferotemporal	82.89 ± 0.75	83.05 ± 0.78	79.54 ± 0.99	0.008
Inferior	80.88 ± 0.76	81.13 ± 0.73	77.26 ± 0.88	0.001
Inferonasal	83.27 ± 0.79	83.61 ± 0.79	79.07 ± 1.08	0.001
Superonasal	85.73 ± 0.82	86.12 ± 0.82	80.46 ± 1.45	<0.001

DM group 1 = patients with type 2 diabetes < 10 years, DM group 2 = patients with type 2 diabetes ≥ 10 years. Values in boldface (*p* < 0.05) are statistically significant. All values are expressed as the mean ± standard errors (μm).

**Table 3:** Superficial macular vessel density in each group using optical coherence tomography angiography.

	Control	DM Group 1	DM Group 2	*p*-Value
Full area	20.32 ± 0.15	19.46 ± 0.17	18.46 ± 0.23	<0.001
Inner area	21.70 ± 0.14	20.77 ± 0.17	19.78 ± 0.24	<0.001
Central area	9.41 ± 0.28	9.34 ± 0.30	8.27 ± 0.31	0.027
Sector				
Superior	21.58 ± 0.19	20.65 ± 0.22	19.72 ± 0.30	<0.001
Temporal	21.62 ± 0.14	20.76 ± 0.19	20.05 ± 0.23	<0.001
Inferior	21.74 ± 0.15	20.75 ± 0.22	19.89 ± 0.30	<0.001
Nasal	21.83 ± 0.22	20.91 ± 0.21	19.49 ± 0.39	<0.001

DM group 1 = patients with type 2 diabetes < 10 years, DM group 2 = patients with type 2 diabetes ≥ 10 years. Values in boldface (*p* < 0.05) are statistically significant. All values are expressed as the mean ± standard errors (mm^−1^).

**Table 4:** Univariate and multivariate linear regression analyses determining factors associated with superficial macular vessel density in patients with type 2 diabetes.

	Univariate		Multivariate	
	B (95% CI)	*p*-Values	B (95% CI)	*p*-Values
Age	−0.01 (−0.03–0.03)	0.997		
Sex	−0.33 (−0.77–0.11)	0.138		
DM duration	−0.07 (−0.11–−0.03)	0.002	−0.05 (−0.09–−0.01)	0.037
BCVA	−5.13 (−9.69–−0.57)	0.028	−5.39 (−9.62–−1.15)	0.013
SE	0.02 (−0.19–0.23)	0.825		
IOP	0.04 (−0.06–0.14)	0.442		
Axial length	−0.03 (−0.39–0.33)	0.866		
HbA1C	−0.27 (−0.56–0.02)	0.069	−0.06 (−0.35–0.23)	0.666
CMT	0.01 (−0.02–0.02)	0.989		
pRNFL	0.02 (−0.01–0.05)	0.096	−0.02 (−0.05–0.01)	0.226
GC-IPL	0.08 (0.05–0.12)	<0.001	0.07 (0.04–0.11)	<0.001

BCVA = best-corrected visual acuity; SE = spherical equivalent; IOP = intraocular pressure; DM = diabetes; CMT = central macular thickness; pRNFL = peripapillary retinal nerve fiber layer; and GC-IPL = ganglion cell-inner plexiform layer. Values in boldface (*p* < 0.05) are statistically significant.
